# A career in bioactive lipids

**DOI:** 10.1016/j.jbc.2025.111041

**Published:** 2025-12-11

**Authors:** Yusuf A. Hannun

**Affiliations:** Departments of Medicine, Biochemistry, Pharmacology, and Pathology and the Stony Brook Cancer Center, and The Northport Veterans Affairs Hospital, Northport, New York, USA

**Keywords:** sphingolipids, ceramide, protein kinase C, sphingomyelinase, cell regulation, enzyme mechanisms

## Abstract

Sphingolipids, first isolated in the mid-19th century, were named as such because of their enigmatic nature. Our lab work over more than 4 decades has attempted to define biologic and mechanistic foundations for this class of lipid molecules. This reflections article recounts my scientific journey as a researcher, beginning from my early years as a Palestinian born in Saudi Arabia who grew up in Beirut, Lebanon, during the tumultuous civil war. Driven by a desire for research and academics, my wife, Lina Obeid, and I relocated to the United States for further training at Duke University in 1983. My initial studies focused on PKC, leading to foundational work on its regulation by lipids. A pivotal discovery was the inhibitory effect of sphingosine on the enzyme, which sparked my interest in sphingolipids as potential bioactive molecules. This anchored my independent career at Duke in 1987, toward the then-underappreciated field of sphingolipid metabolism, leading to a series of groundbreaking findings throughout the 1990s, including the revelation that several sphingolipids, especially ceramides, act as bioactive molecules, with myriad biologic functions. My lab, often in collaboration with that of Lina Obeid, also defined key genes and enzymes in the sphingolipid pathway, fundamentally advancing the understanding of the many roles sphingolipids play in cell biology and disease. The manuscript weaves in some anecdotes and reflections in an attempt to convey to students some of the “hidden” thoughts that operate in the scientific process and the conduct of research.

## Education, medicine, and (teasing) exposure to research (1955–1983)

I believe that every person will have a unique and interesting trajectory. Mine is no exception, though it might differ in significant ways from most American-born scientists.

I was born in Saudi Arabia to Palestinian parents. We moved to Beirut, Lebanon when I was five (1960). My father was a British-educated physician who had served with the British army in World War II (including landing on Normandy). His medical education allowed him to survive and even thrive, after he was forced to abandon his hometown of Jaffa when the city was taken over by Zionist militias in 1948. My mother was very intelligent and excelled at math. She too fled Jaffa in 1948, and her education was cut short. She married my father in Beirut, Lebanon and worked in the home, raising my siblings and me and managing the house.

At the time, Beirut was a wonderful, lively, and peaceful city, often referred to as the “Paris of the Middle East.” I received all my primary education at the International College (a school founded by American educators and missionaries), and then did my undergraduate studies and medical studies and training at the American University of Beirut (AUB), also founded by American missionaries and philanthropists in 1864. AUB has a picturesque campus, with spectacular views overlooking the Mediterranean. At the time, it was probably the best university in the region and attracted students from all over the Arab world and beyond. I started university in 1974, 1 year before full-fledged civil war broke out in Lebanon that lasted until 1990. Nevertheless, college was a stimulating experience, full of possibilities for learning. I discovered my passion for both biology and math and might have considered pursuing a degree in one of these fields, but medicine was one of few viable career paths in Lebanon at the time, so I enrolled in the “premed” program. This allowed me to enter medical school directly after my junior year in college. For a year, I also enrolled simultaneously in the Lebanese University, studying Math and Physics, as my supervisor at AUB initially refused to let me take extra credits in those subjects. But as soon as the restriction was lifted, I undertook a major curriculum in Math at AUB. By the time I entered Medical School in 1976, the civil war had intensified. I spent most of the time in the hospital, which, despite being near the frontlines, also functioned as a safe haven because it catered to all casualties and was respected by all parties.

The intensity of the civil war would wax and wane with frequent ceasefires and relatively peaceful interludes, and we learned how to navigate the exigencies of war and the need to stay safe. Reflecting on that period, I find it amazing how quickly people can adapt to a deteriorating and even degrading environment, and how passive they can be about it.

During medical school, I learned biochemistry in some detail. I still recall the discussion on prochirality with aconitase—I guess it resonated with my desire to understand biology at a very detailed molecular level. I was exposed to some bench-side research, which excited me tremendously. I was instantly hooked. Three professors of biochemistry stand out as significant influences on me: Ibrahim Durr who did his own training with Harry Rudney in Cincinnati on the biosynthesis of mevalonate; Usama Khalidi, a renaissance academician with interests in biochemistry, medicine, and history, who could generate interesting and novel ideas at the drop of a hat; and Naji Sahyoun who at the time was starting his career in the United States as a biochemist, having trained with Pedro Cuatrercassas and then moved with him to the fancy research outfit of Burroughs Wellcome in North Carolina’s Research Triangle. Naji would visit AUB at regular intervals and give very contemporary and exciting lectures on various aspects of biochemical research. At one point, he set up an auxiliary research lab at AUB, where I dabbled with some basic research on platelet proteins. The experience was eye-opening, as we had very little exposure to lab-based research. It was also sobering as it indicated how long it could take to make real progress (a theme I will return to).

Medical training at AUB was highly educational; even as medical students we were expected to fully take care of our patients, functioning more like interns. I was exposed to so many facets of medicine while witnessing the introduction of computed tomography scanning, echocardiography, cardiac catheterization, and the list goes on. I stayed at AUB for my residency training, but I already knew that I was much more interested in academic medicine and had a strong desire to perform research.

Throughout the course of medical school, my career plans and interests had started to crystallize. Internal medicine appealed to me as a primary specialty since it involved more analytical thinking and was getting more focused on basic pathophysiologic mechanisms. Within this expansive specialty, I was gravitating to both Hematology and Medical Oncology, which are often consolidated into one discipline. Hematology was—by the early 1980s—firmly based on biochemistry (*e.g.* disorders involving G6PD, pyruvate kinase, complement pathway, hemoglobinopathies, *etc.*) and gaining significant grounding in genetics and molecular biology (*e.g.* molecular basis of sickle cell anemia and the thalassemias with novel discoveries on gene regulation). Oncology, by contrast, was still in the dark ages, based mostly on “trial and error” development of cytotoxic therapeutics. Thus, it promised major discoveries.

I started to explore training abroad in Heme/Onc and zeroed in on the United States for the research possibilities and state-of-the-art training. Naji Sahyoun helped me obtain a visiting observership at Duke University in North Carolina, where I spent 3 months in the Spring of 1982. Duke exuded professionalism, hard work, and academics: Duke residents and faculty were dubbed “Duke Marines.” I spent two of those months serving on clinical rounds, where I was taken under the wings of Hal Silberman, an amazingly observant, compassionate, and competent oncologist. I also spent 1 month in the laboratory of Russ Kaufman studying globin gene regulation. Incidentally, in April of 1982, UNC won the men’s NCAA basketball championship, and Chapel Hill erupted into a frenzy. It was a novel experience for me!

Upon returning to Beirut, I focused on my residency training. But at the same time as my career goals were maturing, the political situation in Lebanon was deteriorating. The country had fallen into severe interfactional violence, and Israeli and Syrian occupations brought new levels of violence to Beirut, including the protracted Israeli siege of the city in the Summer of 1982. Throughout the civil war, car bombs exploded randomly all over the city. At the hospital, we would treat the victims.

As a Palestinian, it became obvious that I needed to find a new home. This sealed the desire to immigrate permanently to the United States, and I applied to several fellowship programs for subspecialty training in Hematology and Medical Oncology. I was accepted at Stanford (conditionally) and at Duke where I was a known commodity.

By that time, I was engaged to Lina Obeid, who I had met in high school, although we did not get together until medical school. She also visited Duke and applied to the residency program there. With Lina’s qualifications and forceful personality, she was promptly accepted, so we made our joint plans to relocate to the United States and continue our training at Duke. Lina and I were married in April 1983, and we completed our medical school and residency training, respectively, in June, the same month we left for the United States. It was the start of a long and wonderful marriage and work collaboration.

## Medical training at Duke (1983–1986)

Arriving in Durham, NC, we had to simultaneously adapt to the United States, set up our home, and figure our way into our new jobs: Lina as an intern and resident in Internal Medicine, and myself as a fellow in Hematology and Medical Oncology. Although Lina was born in New York City and had done most of her undergraduate studies at Rutgers in the late 1970s, she had spent the majority of her life until that point in Beirut.

The culture shock was substantial. My primary image of the United States had come from American movies and literature, and here, we were thrust into the US south, where “foreign” did not apply only to us but to anyone from anywhere else in the country. Compared to Lebanon, I found the political scene timid at the time. In the Middle East, there was a vibrant, and at times violent, political scene that spanned the spectrum from diehard communism to radical right-wing fascism to Islamism—no wonder it took me a while to figure out the differences between democrats and republicans.

As I mentioned, Duke was invigorating and demanding. Back then, there were no limits on hours worked per week for residents and fellows, and we spent most of our time in training. My first year was totally devoted to patient care and teaching. However, starting with the second year, I had an expanding opportunity to participate in research. In those “golden days” of academic clinical medicine, subspecialty training included a substantial amount of protected time for research. At Duke, some of my senior fellows opted to do lab-based studies, which is what I was very eager to do—a path made easier by the culture at Duke.

## Research training at Duke (1984–1987)

My first rotation in the lab in 1984 was with Jim Niedel, then an up-and-coming junior faculty who had just made a splash by publishing a study showing that PKC was indeed the elusive receptor for phorbol esters (Jim later became the senior VP for research and development at Glaxo-Wellcome).

### Historical perspective, PKC

To put this into the historical context of biomedical research, I was thrust into the opening salvo of what was rapidly emerging as the discipline of signal transduction. The field’s origins started with phenomenal discoveries in the 20th century of hormones, their receptors, and their actions. Subsequent pioneering studies on cAMP, its target kinase PKA, and roles of protein phosphorylation were defining how events at the plasma membrane (PM, *e.g.* hormone binding to receptors) translated into a signal to the cell (cell signaling). In the late 1970s, Nishizuka’s group in Kobe Japan identified a new protein kinase in the brain that was activated by proteolysis (1). In a series of clever and rigorous studies, the group made a seminal discovery that this kinase (now named PKC) was activated by calcium, phospholipids, and neutral lipids, especially diacylglycerol (DAG) (2, 3, 4). These groundbreaking discoveries identified a second regulated kinase AND connected it to lipid metabolism. The latter was based on pioneering studies by Hokin and Hokin in the 1950s, where they discovered that hormone action resulted in acute turnover of membrane phospholipids, especially phosphatidylinositol (PI) (5), a process termed the PI cycle (6). Since DAG was a metabolic intermediate in this cycle (direct breakdown of PI to DAG which then served as a substrate for the resynthesis of PI), Nishizuka’s work connected PKC to the PI cycle. Not stopping there, Nishizuka’s group, in collaboration with Monique Castagna, showed that phorbol esters (xenobiotics from Euphorbiaceae plants) also directly activated PKC (7). It was another phenomenal discovery. In the 1960s and 1970s, extensive cell biology literature had accumulated on the various and potent effects of phorbol esters on cell function (*e.g.* tumor promotion, differentiation, metabolic effects, migration, growth, *etc.*). Mechanistic studies had shown the existence of a receptor for phorbol esters, but its identity was elusive. Nishizuka’s discovery suggested that perhaps PKC does indeed function as the receptor for phorbol esters, and that is what the Niedel group formally established (8).

### First “real” experiments

At the time, the emerging connection of PKC to DAG was puzzling to lipid biochemists as to how such a critical intermediate in phospholipid metabolism could function as a second messenger. This was not the case for cAMP which does not have a comparable metabolic role. (Spoiler alert: it became clear with subsequent studies that PKC was primarily activated at the inner leaflet of the PM where it bound phosphatidylserine and became activated by DAG in this location.) This question started a collaboration between Niedel and Robert (Bob) Bell, who at the time was an assistant professor of biochemistry at Duke, where he was already making solid and seminal contributions to the study of phospholipid metabolism. In 1984, I joined the Bell lab to study PKC. The lab was medium to large in size (perhaps 12–15 graduate and postdoctoral students), but the atmosphere was electric. I learned so much from a host of interesting projects being carried out by many talented students. In particular, Carson Loomis, a postdoc at the time and a previous trainee in the Tanford lab, took me under his wings; from him I learned how to conduct precise experiments in various aspects of lipid and protein biochemistry. Ironically, my first project was geared at disproving the regulation of PKC by DAG. Bob had the idea that Nishizuka was perhaps copurifying DAG kinase (DGK) with PKC and that they were measuring the production of phosphatidic acid (PA) in addition to the phosphorylated histone substrate. It was a plausible hypothesis, worth investigating: at the time, Nishizuka had probably published only one activation curve of PKC by DAG (3), which did not show saturation, lending more credence to the DGK/PA possibility (*i.e.* the more DAG is added, the more PA is formed). I suspect Nishizuka was not happy with this result, and almost all subsequent figures showed the effects of DAG on the calcium dependence of PKC (which avoids the possible issue of production of PA). In my studies, I could purify PKC to a satisfactory level and separate it from DGK. For an assay, I adopted a mixed micellar approach for studying regulation of PKC by DAG, adapted from work by Ed Dennis (9) on using mixed micelles for the study of phospholipase A2 (10). These results netted me my first biochemical research paper—published in *Journal of Biological Chemistry* (JBC, (11))! I continued my work on PKC in Bob’s lab and published several papers (all in JBC). *Anecdote*: JBC was “it” back then, the main target journal for any self-respecting biochemist. I remember one postdoc from Japan coming to the Bell lab, working very hard, and producing a great paper. Bob wanted to send it to *Cell*, but the postdoc became dejected: he had been instructed by his mentors in Japan to go to Bob’s lab and get a JBC paper!

I then obtained a K award from the National Institutes of Health (NIH) in 1985 that allowed me to extend my fellowship in the lab. I was working exceptionally hard, driven by the excitement of the work and also by the fact that Lina, in her residency years at the time, often had long weekends in the hospital (before the 80 h/week was instituted as a limit for residents). I recall once struggling with a new assay I was developing for phorbol ester binding to PKC using the mixed micelles approach. I resolved the technical issues on Friday. By Monday evening, working almost nonstop, I had obtained at least an n = 1 for many of the results. This research also ended in a JBC paper (12).

**Figure 1:**
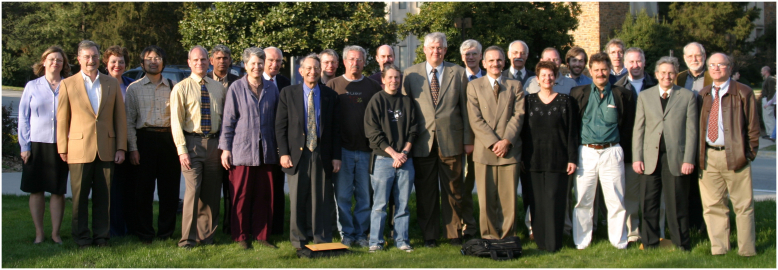
**Robert Bell in *center* with lab graduates, colleagues, and friends at celebration of 60th birthday.** From *left to right*; Mayre Loomis; Carson Loomis; Linda Karolak; Toshiro Okazaki; Thomas Mcintyre; Wasiuddine Khan; Sue Gidwitz; John Exton; Ed Dennis; Phil Green; Larry Ballas; David Burns (*back*); Elaine Bardes; Robert Bell; Jim Walsh; Yusuf Hannun; William Dowhan; Rosalind Coleman; not identified; Ben Buehrer; Andrew Quest; Timothy Larson; Christopher McMaster; George Carman; Alfred Merrill; and Christian Raetz.

Bob is a very rigorous scientist and extremely careful in what he published. He often made sure someone else in the lab was able to obtain the same key results before sending in a paper. From him and the group (and attending graduate biochemistry lectures), I learned lipid biochemistry, protein purification, enzymology, and thermodynamics. Figure 1 shows Bob and several of his scientific colleagues and trainees at his 60th celebration in 2004.

## Sphingosine, an Off-The-Shelf Discovery

While in Bob’s lab, Carson Loomis and I did a major screen of the effects of various lipids on PKC. Back then such studies were dismissively labeled as “fishing expeditions,” studies without a clear anchoring hypothesis. Now, we call them “unbiased approaches.” The idea, however, was not entirely random: since PKC was regulated by at least two lipids (phosphatidylserine and DAG), could it be regulated by more lipids? We collected all the lipids present in the Bell lab and tested them on PKC. They were all duds with the exception of a molecule called sphingosine. No one in the lab knew what it was or why it was even on the shelf in the first place (in hindsight I suspect it may have been left by Al Merrill who did a 1-year postdoc in Bob’s lab a few years before I joined). The effects of sphingosine were quite specific from a lipid point of view, and as regulation of PKC by lipids went, also potent (13). Unsure of what to make of the results, Bob discussed them with Al Merrill, who did some studies to confirm and extend the results, initiating a collaboration (14). I also demonstrated the ability of sphingosine to inhibit PKC in platelets and then eukaryotic cells. These results spawned a plethora of studies with sphingosine. Many investigators who had worked with phorbol esters in cell biology evaluated the effects of sphingosine. The results were consistent with inhibition of PKC.

More critically, these results made me start thinking about sphingolipids and imagining possibilities of regulated metabolism of sphingolipids. Specifically asking: are the levels of any sphingolipid regulated? Is there at least one enzyme of sphingolipid metabolism that is regulated? At the time it sounded fanciful, but it became the main focus of my own lab as I set it up at Duke in the Department of Medicine (Hematology and Medical Oncology).

### Historical perspective, the sphingolipids

**Figure 2:**
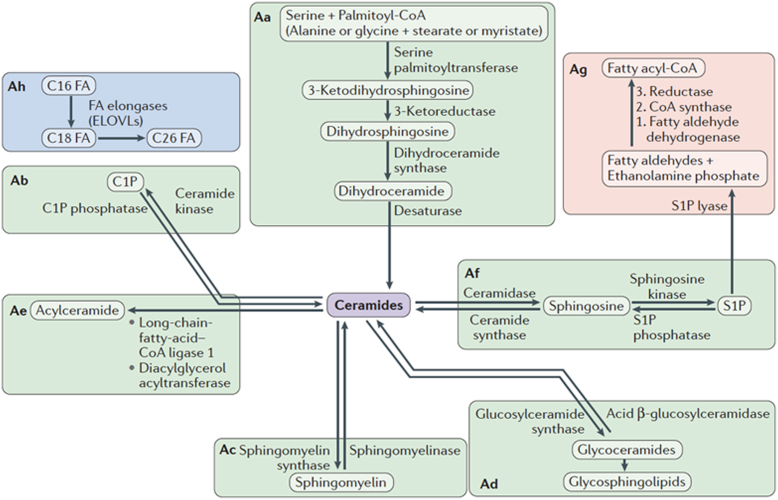
**Connected metabolic modules.** The figure shows in simplified mode the main metabolic modules that constitute the “basic” pathways of sphingolipid metabolism. *Aa*, the *de novo* synthesis module is the “entry” into sphingolipids whereby the first identifiable sphingolipid, 3-ketodihydrosphingosine, is formed by the condensation of an amino acid (mostly serine but also alanine or glycine) and a fatty acid (mostly palmitate but also myristate and stearate). *Ab*, Phosphorylation of ceramide to C1P and dephosphorylation of C1P. *Ac*, formation and hydrolysis of SM. *Ad*,. formation and breakdown of glycosphingolipids. *Ae*, formation of 1-O-acylceramide. *Af*, hydrolysis of ceramide to sphingosine and formation of S1P (and reverse reactions). *Ag*, exit from sphingolipids through cleavage of S1P. *Ah*, fatty acid elongation, which generates very long–chain fatty acids that are mostly targeted for incorporation into sphingolipids. Figure reproduced from (20); and please refer to this reference for a more detailed discussion. S1P, sphingosine 1-phosphate.

The usual texts of biochemistry in the 1970s and 1980s had at most a page (out of hundreds) on sphingolipids, and mostly in the context of lysosomal storage disorders (Niemann-Pick for sphingomyelin (SM) and Gaucher for glucosylceramide). The recurrent assessment in these textbooks was that sphingolipids are “inert” molecules, a kiss of death for spawning interest. Delving into the primary literature, however, I came across several important aspects of these molecules. They were first discovered by Johann Thudichum, a German physician scientist working in England. His interests were varied—he wrote on wine and cooking and invented medical instruments such as the nasal speculum. But his main focus was on the chemistry of the brain, which led him to isolate a nondissolvable lipid that he called “sphingosin,” named because its enigmatic nature invoked the riddle of the Sphinx (15). Pioneering work by Herbert Carter and others starting in the 1930s elucidated the structure of sphingosine and a few other sphingolipids (16) and led to a rudimentary definition of the synthetic pathways. Work by Hakomori and others starting in the 1950s on glycosphingolipids revealed the complexity of their structure and their roles as antigens and protein ligands/cofactors (17, 18). Work by Robert Lester advanced our understanding of synthesis of sphingolipids using the yeast *Saccharomyces cerevisiae* (19). Still, there were hardly any studies on sphingosine, let alone ceramide or sphingosine 1-phosphate (S1P). These molecules were relegated to the role of intermediates in the metabolism of more complex sphingolipids such as SM and the glycosphingolipids (Fig. 2 shows a contemporary depiction of sphingolipid metabolism).

## Starting the Lab at Duke (1987–1998)

Having obtained my first NIH grant (K award), and having published a few papers in JBC, I was offered a faculty position at Duke staring in July 1987. As fate would have it, Lina was pregnant at the time, and she delivered our triplets in September of that year. These were exciting but very hectic times for Lina and me both at work and at home, as we tried to juggle all of our responsibilities. The upside of that is having the triplets focused me on being more selective and careful with research projects.

One of the main decisions to make in setting up a lab is whether to put several trainees on the same project so that it moves faster or to develop a specific project for each trainee. There are obvious pros and cons for each, and it is important for scientists to ponder and resolve this issue when embarking on their independent careers. For myself, I chose to follow the one trainee-one project path, as it was equally important to me to work on the development of each trainee as on developing a specific project.

I was able to recruit a great team—throughout my career I have been blessed with outstanding trainees. I will not sing their praises individually here, but they are all remarkable. The lab also attracted several visiting scientists, who enriched the environment (Table 1). My first fellow was Toshiro Okazaki, a visiting physician scientist from Kyoto, and his project was focused on discovering the regulation of sphingolipid levels in cells. We first looked “where the light is,” so to speak. We studied effects of different growth factors, looking for changes in SM (the easiest sphingolipid to measure at the time, which could be readily detected by thin layer chromatography [TLC]). But we came up short. Toshiro had studied leukemia cell differentiation in Kobe before coming to Duke, so we turned our attention to regulators of HL-60 leukemia cells. One day, Toshiro came with a TLC result showing dramatic loss of SM in response to vitamin D3. In retrospect, we were lucky because the exposure was just so that it showed a black and white result; upon quantitation, this was about a 20% change. We used this to probe further, and Toshiro discovered larger changes in ceramide and changes in activity of a neutral sphingomyelinase (21). We all became very excited with the results and their prospects. Had we known how long and tortuous the road would turn out to be, we probably would have abandoned the work right there and then. Early on, Alicja (Ala) Bielawska, a chemist and recent émigré from Poland joined the lab, and she quickly became the world’s expert on sphingolipid chemistry. Ala designed and synthesized soluble short chain ceramides (*e.g.* C6-ceramide), which became a mainstay of ceramide cell biology studies. Her work allowed us to demonstrate that ceramide was bioactive. It induced differentiation of leukemia cells. Interestingly, dihydro C6-ceramide, which only differs from C6-ceramide by lacking the 4 to 5 double bond, was inactive in those biologic studies (22), reassuring us that the effects of ceramide were highly specific. Mie-young Kim who joined the lab as a visiting professor from South Korea was able to demonstrate that tumor necrosis factor (TNF) also induced SM turnover and ceramide formation, extending the significance of the work (23). Richard Kolesnick’s group showed that phorbol esters induced SM hydrolysis (24).

**Table 1:** Mentees and visiting scientists

Postdoctoral ostdoctoral fellows					
Toshiro Okazaki	1988–1991	Charles Chalfant	1997–2001	Mohamad El-Osta	2007–2012
Mark Werner	1987–1991	Paola Signorelli	1997–2001	Daniel Canals	2008–2015
Alicja Bielawska	1987–1994	Hirofumi Sawai	1998–2000	Delphine Milhas	2008–2009
Wasiuddin Khan	1989–1993	Hanna Suidan	1998–2000	Michael V Airola	2010–2017
Robert Wolff	1990–1994	Chiara Luberto	1998–2002	Monica Garcia-Barros	2011–2019
Rick Dobrowsky	1990–1995	Yasuo Okamoto	1999–2002	Masa Wada	2011–2015
Ghassan Dbaibo	1991–1996	Lauren Ashley Cowart	2001–2005	Nadia *Rana*	2012–2015
Marina Pushkareva	1991–1994	Norma Marchesini	2001–2004	Nicolas Coant	2012–2018
Adrienne Richards	1993–1995	Silvia Vaena de Avalos	2001–2006	JP Truman^d^	2012–2022
Nejemie Alter	1993–1996	Fernando Alvarez	2001–2009	Alice Rutatangwa	2014–2016
Bin Liu	1994–1998	Kazuyuki Kitatani	2003–2009	Mohamed Salama	2015–2018
David K. Perry	1994–1998	Jeff Jones	2003–2005	Cosima Rhein	2015–2016
Futoshi Nara	1994–1996	Bill Wu	2004–2012	Magali Trayssac^d^	2015–2022
Tim Driscoll	1994–1998	Jola Idkowiak-Baldys	2004–2010	Jihui Ren	2015–2021
Kellie Rizzieri	1996–1998	Hiroshi Kitagaki	2005–2007	Bachar Hassan	2016–2021
Supriya Jayadev	1995–1996	Christopher Clarke	2005–2011	Evgenii Boriushkin	2019–2022
Sheree long	1995–1997	Motohiro Tani	2005–2007	Fabiola Velazquez^d^	2016–2021
Sandra Merrick	1995–1998	Nabil Matmati	2006–2015	Botheina Ghandour	2021-
Zdzislaw Szulc	1997–2003	Nana Bartke	2007–2009	Masayoshi Higuchi	2021-
Samer El-Bawab	1997–2001	David Montefusco	2007–2012	Nihal Medatwal	2022-
				Mojtaba Sadeghi	2025-
Ph.D. students					
Corinne Linardic	1989–1993	Jeff Jones	1998–2003	Benjamin Newcomb^MSTP^	2011–2016
Supriya Jayadev	1990–1995	Kevin Becker^MSTP^	1999–2003	Achraf Shamseddine^MSTP^	2011–2015
Gerard Blobe MSTP	1991–1994	Ben Pettus^MSTP^	1999–2003	Justin Snider	2012–2019
Julie Fishbein Saba	1991–1996	Yousef Zaidan^MSTP^	2004–2007	Prajna Shanbhogue	2014–2019
Heather Hayter	1994–1999	Russell Jenkins^MSTP^	2006–2010	Wataru Sakamoto^j^	2015–2017
Gary Jenkins	1994–1998	Mikael Pata^l^	2006–2007	Samia Mohammed	2015–2021
Jiandi Zhang^d^	1994–1998	Aintzane Apraize^c^	2007–2008	Joseph Bonica^d^	2015–2022
Xiao Fen	1995–1998	Vinodh Rajagopalan	2008–2014	Allen Lee MSTP	2018–2024
Namjin Chung^d^	1995–1999	David M. Perry^MSTP^	2008–2012	Mojtaba Sadeghi	2019–2025
Sun Sik Bae^b^	1996–1997	Fabio Simari^f^	2009–2010	Andrew Resnick^g^	2020-
Chiara Luberto^e^	1996–1998	Mengling Liu	2010–2016	Sam Chiappone	2021-
Paola Signorelli^h^	1997–1999	Alessandra Metelli^i^	2011	Khalayi Martha Aywa^a^	2022-
Katsuya Kishikawaj	1998–2001	Po-Wei Chen^k^	2011	Sagarika Chowdhury	2023-
Research medical students (Duke)					
Richard Chao	1991–1992	Mark Yeh	1994–1995	Carsten Sorensen	1996–1997
Fausta Nazaire	1992–1993	Chris Gamard	1994–1996	David Yu	1997–1998
Visiting scientists					
Mie-young Kim	1989–1990	Ignacio Hernandez	1997	Julnar Usta	2000–2001
Maija-Liisa Rasilo	1993	Claudia van Thiel	1997	Talat Saatov	2002
Sehamudin Galadari	1994–1996	Katsuya Kishikawa	1997	Sehamuddin Galadari	2000–2003
Pan-Ghill Suh^b^	1995–1996	Bart-jan Kroesen	1998–1999	Anna Maria Porcelli	2002–2003
Wendy boss	1997	Hannie Sietsma	1998–1999	Giovanni D’Angelo	2016
Thomas Weider	1997				

The table represents the list of graduate and postdoctoral students in the lab. Also included are the visiting scientists. I apologize for not including the names of the dozens of high school and undergraduate students (more than 150), who came to the lab; many of whom enriched the work.

MSTP, Medical Scientist Training Program.

a Joint with Mike Airola.

b Pohang University, Korea.

c University of the Basque Country, Bilbao.

d Joint with Lina Obeid.

e Catholic University, Rome.

f Institute for Advanced Chemistry of Catalonia, Barcelona.

g Joint with Chris Clarke.

h University La Sapienza, Rome.

i University of Perugia.

j Ono Pharmaceuticals.

k Georgia Tech.

l University College Dublin.

### Historical perspective on growth suppression and apoptosis

Our work on sphingolipids at Duke during the 1990s continued to develop and was starting to attract attention of several investigators. Those were very exciting times for us, despite the prevailing attitudes, which frowned upon the study of TNF and other suppressors of growth. Such agents were dismissed as “chalones,” so we were swimming against the current. Going against prevailing attitudes became worse when we connected to apoptosis. By that time, Lina was starting her own independent work, after having spent a couple of years in my lab. In our previous studies, we had noticed that C6-ceramide would become quite toxic at slightly higher concentrations than those needed to induce differentiation. Given the biases against such studies, we avoided pursuing the growth suppressive effects and restricted our work to regulation of differentiation. However, by a lucky convergence of key scientific discoveries in the 1980s, the fields of growth suppression and cell death were thrust into the limelight. Although apoptosis as a biologic phenomenon was described in the 19th century and resurrected in the 1960s and 1970s (25), it entered the mainstream in the 1980s, especially with the finding that the Bcl2 oncogene functioned by inhibiting apoptosis (26). At the same time, the retinoblastoma gene product, Rb, was discovered as a tumor suppressor (27). Likewise, WT p53 was found to also function as a tumor suppressor, inducing growth arrest and cell death (28). Also, TNF was upgraded/clarified from a TNF to an inducer of apoptosis along with identification of “death domains” in its receptors and other related receptors. These studies and our novel connection between TNF and ceramide prompted us to investigate the role of ceramide in apoptosis.

### Hooked on sphingolipids

The results obtained by Lina in the lab with help from Corinne Linardic were astounding. Exogenous ceramides caused profound apoptosis, especially in lymphocytes (29), which we later showed resulted in activation of downstream caspases and was prevented by Bcl2 and the related Bcl-x. This enhanced the visibility of sphingolipids dramatically and opened several avenues for research.

In the course of trying to understand and manipulate sphingolipid metabolism, we soon realized that the molecular and biochemical underpinnings of this pathway were seriously deficient. By the mid-1980s, of all known enzymes of sphingolipid metabolism at the time, only the gene encoding acid glucocerebrosidase was cloned (30) after thorough studies on Gaucher’s disease, the identification of accumulation of glucosylceramide, and the identification of the defective enzyme, glucocerebrosidase (31). Therefore, in the early 1990s, we turned to the yeast *S. cerevisiae* as a model organism through which we could study these pathways and define key genes. Serendipitously, my next office and lab neighbor, Steven Garritt, was a yeast molecular biologist, so we started planning some yeast experiments. These resulted in our group first identifying the gene for S1P lyase through the work of Julie Saba with help from Futoshi Nara (32). Lina and Cungui Mao identified S1P phosphatase (33) and the family of alkaline ceramidases (34), whose existence was not even suspected at the time. Namjin Chung, working with Lina and me defined roles for sphingoid bases in yeast in uptake of amino acids (35). We also found that ceramide activates protein phosphatases in yeast (36), and Nickels and Broach independently defined its subunit composition (37). Gary Jenkins discovered the regulation of *de novo* synthesis by heat stress (38) which paved the way for several important inroads into the heat stress response of yeast.

I will not go into details here, but our lab at Duke during the 1990s was making rapid progress along several fronts in sphingolipid biochemistry and cell biology through the efforts of many talented graduate and postdoctoral students. Very briefly, Rick Dobrowsky connected sphingomyelinases and ceramide to the neurotrophin family (39). Ghassan Dbaibo, Marina Pushkareva, Supriya Jayadev, and Bob Wolf identified the regulation of Rb, myc, and cell cycle by ceramide (40, 41, 42, 43), whereas David Perry, Myriam Smith, Jiandi Zhang, Heather Hayter, Paola Signorelli, and Ghassan Dbaibo demonstrated regulation of caspases and p53 (44, 45, 46, 47). They also showed that Bcl2 acted downstream of ceramide to prevent induction of apoptosis, whereas work by Dbaibo showed that upstream but not downstream caspases were necessary for generating ceramide in response to apoptotic stimuli (48). We (Linardic) began to identify possible distinct intracellular sites for action of ceramide (49). Biochemically, Bin Liu was able to purify neutral sphingomyelinase to near homogeneity and characterize it biochemically (50). This was critical for us as the prevailing dogma held that since there is already acid sphingomyelinase, there is “no need for another sphingomyelinase.” We showed its activation by oxidative stress as well as by several inducers of apoptosis (51). Importantly, Rick Dobrowsky discovered the ability of ceramide to activate protein phosphatases *in vitro* and relate that to regulation of protein dephosphorylation in cells (52), leading to several studies by us and others implicating phosphatases in the cellular actions of ceramide. Chiara Luberto, a graduate student from Italy at the time, conducted elegant studies on regulation of mammalian SM synthase by oncogenes. In the chemical biology arena, we developed, in addition to the soluble ceramide analogs, inhibitors of ceramidases, stereoisomers of ceramides and sphingosines, and other related molecules (Bielawska and Zdzislaw Schultz).

Ideas in the lab at the time were coming from all directions. I would characterize them in hindsight as ambitious and somewhat scattered, but we were trying to gauge the scope of the field of sphingolipid biology, not knowing how vast it was going to turn out to be. Thus, it seemed that wherever we turned, we made hits, for example, connecting sphingolipids to growth, differentiation, and apoptosis, then inflammation, senescence *etc.* In hindsight, we were shooting fish in a barrel: we now appreciate that the various bioactive sphingolipids are involved in almost all facets of cell biology. It was our period of discoveries in cell biology and in advancing the molecular and biochemical underpinnings of sphingolipid research.

The field was significantly enhanced by the work of Sara Spiegel on discovering biologic actions of S1P (53, 54) and the work of many others on additional bioactive sphingolipids and their enzymes.

### But continuing work on PKC

One strategic decision I made when I started my lab was to maintain our studies on PKC, albeit in a limited fashion. The rationale was that the PKC field was much more advanced than that of sphingolipids and provided us with more biochemical and molecular biology tools to work with (*e.g.* protein purification, enzymology, developing antibodies to purified proteins, availability of cDNA, constructing GFP fusions, *etc.*). My motivation was to retain and expand on these areas of expertise in the lab, while we tackled distinct approaches with sphingolipids (*e.g.* enzyme purification, lipid isolation, TLC, lipid labeling in cells, yeast genetics, synthesis of sphingolipid analogs and enzyme modulators). Looking back, this was rewarding at two levels. First, we continued to make inroads on PKC (*e.g.,* advanced kinetic analysis of regulation of PKC by its cofactors and activators (55) and the regulation of transcription of PKC (Obeid)) (56). Gerry Blobe studied selective formation of PKCß1 and ß2 (57), and defined isoform specific functions in cells (58, 59). Xiao Feng demonstrated visually and conclusively the translocation of GFP-PKC to the PM and mechanisms of regulation of this association (60). Feng also studied the autophosphorylation of PKC and its role in reverse translocation of PKC (61). A very talented and hard-working postdoc, the late Wasiuddine Khan, advanced the studies on regulation of PKC and on its roles in platelets (62, 63). The latter was further developed by Mark Werner, focusing on signaling by thrombin (64). Second, the sphingolipid field started requiring these approaches as it developed with identification of genes for the various enzymes. Moreover, when I was appointed in Hematology, I was designated as the “platelet expert” based on a couple of studies I had published using platelets. These were enabled by a strong collaboration with Charles Greenberg, who was the real expert in hemostasis/thrombosis and platelets.

### Career development

Career wise, both Lina and I were advancing in rank with myself receiving the Wayne Rundles Endowed Chair in Oncology at Duke. Our publications were receiving high attention, and our funding was solid but a constant struggle as a majority of scientists outside the field were simply unfamiliar with it. I was also experimenting with some leadership positions (*e.g.,* Director of Program in Molecular Medicine, Interim Associate Director for Research at the Duke Cancer Center). The triplets were in elementary school, and we had structured our family life around them. Lina and I took turns in getting home early enough to take care of the children and prepare dinner. I had Tuesdays and Thursdays, and my dinners were basic at best. Lina had the remaining weekdays, and she would invariably whip up an amazing meal.

## Advancing the research in Charleston (1998–2012)

### Moving to Charleston

One Summer in the early 1990s, returning from a family beach vacation in Hilton Head, we stopped to explore Charleston, a historical city that had not yet achieved its current vast national recognition. We were taken by it. The city was still rebuilding after hurricane Hugo (1989), but it was attractive. We wondered about working there some day (I must confess, neither of us had heard of the Medical University of South Carolina, MUSC, at the time). In the mid-1990s, a colleague and friend of ours from Duke, Ian Taylor, was recruited to MUSC as Chair of Medicine, and he kept telling us about how great the university was becoming. He tried to recruit us a couple of times. When he told me the position of Chair of Biochemistry was open, I grew intrigued, as my favorite part of our research has always been its biochemical underpinnings. Moreover, I realized that I did enjoy recruiting, building programs, and mentoring junior faculty. In 1998, Lina and I moved our family and our laboratories to Charleston. [A note to those weighing such decisions: I find that there is an intellectual tradeoff between focusing totally on one’s lab *versus* building a program with other faculty. In one’s lab, the PI is responsible for the entire scope of the research, from buffers and methods to organizing projects, to strategizing on goals (and throw in some business administration, fund raising, counseling activities, HR, PR, and whatever other Rs one can think of. Of course, there are additional obligations in leadership roles, and building a strong leadership team is critical). In working with junior faculty, the focus is almost totally on the strategy and development of a project. The tradeoff: you engage in highly exciting thinking with other faculty, but you do not get your name on their papers.]

### Leadership roles

I joined a department that needed rebuilding as there had been hardly any new recruits for years, and there were only a handful, but really good, cadre of active and funded investigators. I was given a relocation package for my group—which I insisted should be separate from the department’s—and a $2 million package to recruit as many faculty as I could. (Today, this could be expended on a single recruitment). I also negotiated getting part of the indirect costs and all the salaries recovered on grants, which proved instrumental down the road by generating substantial income for the department, allowing it to continue growing. Within a few years, we (myself, the new recruits, and the existing faculty at MUSC who all rose to the occasion), built a thriving department. Our NIH ranking in funding galloped from the low 80s to number 18. My advice to faculty starting their independent careers has been to focus on the science and their passion (and not fashion) in research. My analogy is that a starting faculty has to put wheels on a cart so that they can move it and gain momentum. The wheels (read: subjects of grants, papers to write, projects to assign, and areas to explore) should all point in the same direction. Otherwise, it is very difficult to generate momentum.

But it was not just our department that thrived, the leadership at MUSC at the time (James Edwards, Layton McCurdy, Rosalie Crouch, Ray Greenberg, Jerry Reves, John Raymond) was totally focused on advancing academics, and within a few years, MUSC’s NIH national ranking jumped to 42nd. In a personal dark spot on those otherwise electrifying early days at MUSC, a few months after moving to Charleston, I lost my father to lung cancer.

Within a year of my arriving at MUSC, I was tapped to serve as Deputy Director of the Hollings Cancer Center (named after Senator Fritz Hollings, a wonderful human being and an ardent supporter of MUSC). We developed three very robust research programs that ultimately earned us National Cancer Institute (NCI) designation. I also refocused the biologic aspects of our research on cancer-related biology and significance. This led to several productive collaborations and to our acquiring a program project grant from NCI on sphingolipids in cancer (still active).

### Continuing studies on sphingolipids

**Figure 3:**
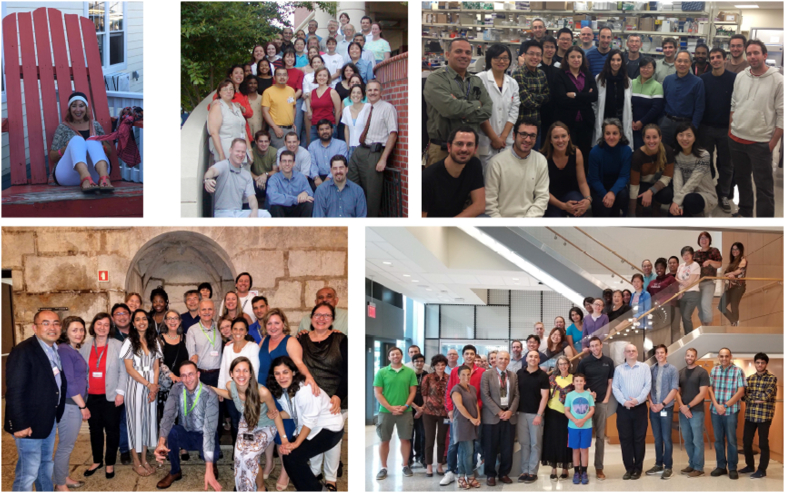
**Lab photos.***Upper left:* Lina. *Upper middle*: the Hannun-Obeid Lab Groups in Charleston in 2004. *Upper right:* Members of the Hannun-Obeid lab groups upon Arrival to Stony Brook, 2012. *Lower right:* The Hannun-Obeid Lab Groups in 2019 in the then newly constructed MART building. *Lower left*: Hannun-Obeid Lab members and friends at the 10th iCC meeting in Cascais, Portugal 2019. iCC, international Ceramide Conference.

**Figure 4:**
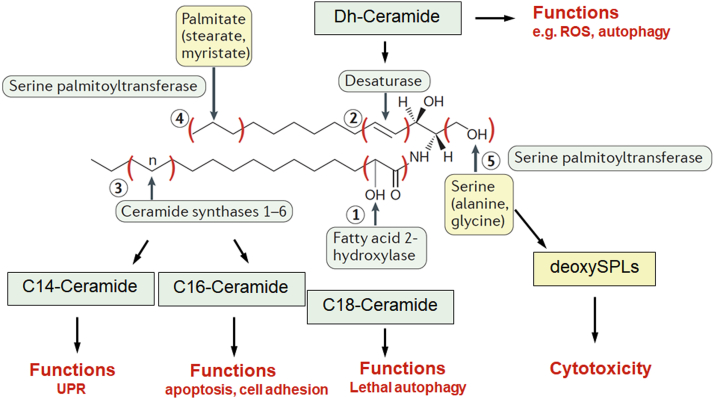
**The ceramide family.** Advanced LC/MS has revealed that “ceramide” is indeed a family of closely related molecules. The figure shows the many structural variations in ceramides, and it is important to note that these variations are introduced or at least regulated by specific enzymes and enzyme components. In conjunction with the multiplicity of the enzymes of ceramide metabolism and their distinct subcellular localization, this has served as the foundation for the many ceramide hypothesis (67). Figure reproduced from (20) with modifications.

**Figure 5:**
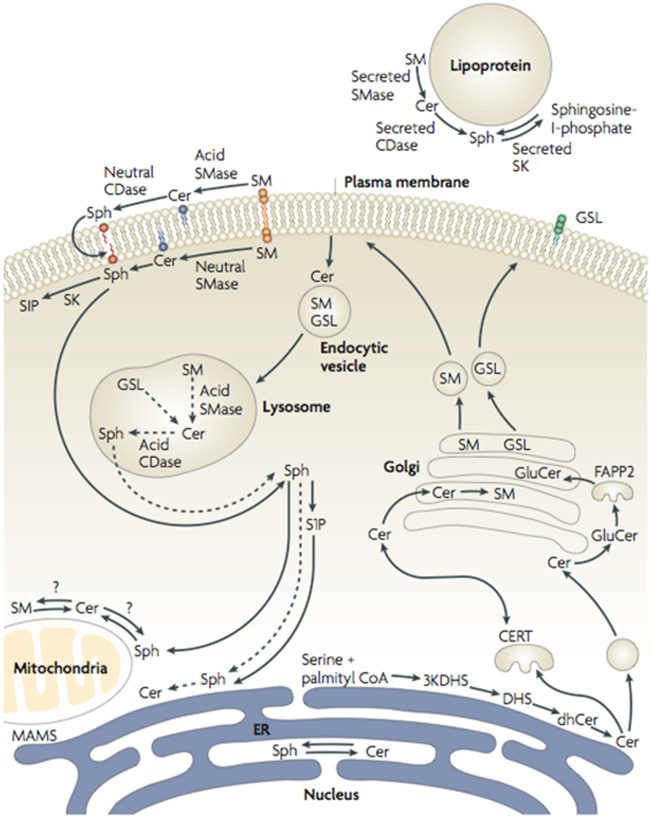
**Subcellular and topological Organization of sphingolipid metabolism.***De novo* synthesis commences in the endoolasmic reticulum (ER) through the formation of ceramide which is then transported to the Golgi (through vesicular trafficking and/or action of the transport protein CERT) where it is converted to SM and/or glycosphingolipids. The latter two constitute the family of “complex sphingolipids” and they are trafficked to the plasma membrane. SM and glycosphingolipids can be endocytosed to the lysosomes where they are catabolized to ceramide and then to sphingosine. It is now appreciated that each major cellular sub compartment harbors its own set of enzymes that act on ceramide and related sphingolipids. Figure reproduced from (68). SM, sphingomyelin.

Most of our students and postdocs moved with us from Duke (Fig. 3, *upper middle* shows the group in Charleston in 2004). Shifting to a basic science department allowed us to develop a lipidomics core focused on sphingolipids, initially through the efforts of MD-PhD student Ben Pettus (65) but then formalized by recruiting Ala’s husband, Jacek Bielawski (66). These were among the very first “lipidomic” activities, and they allowed us to quantitate the lipids of interest. More importantly, we were very quickly introduced to the vast complexity of structure for ceramide (Fig. 4) and other sphingolipids. At the same time, our cloning efforts and those of others revealed another layer of complexity: many of the enzymes of metabolism of bioactive sphingolipids are actually families of enzymes, products of distinct genes. This led to the appreciation that these enzymes reside in distinct compartments (Fig. 5). In turn, these revelations enabled us (eventually) to articulate and propose the Many Ceramides hypothesis, whereby we posited that the regulation and function of bioactive sphingolipids is primarily governed by their subcellular localization, mostly determined by the location of the enzymes that regulate their levels (67). With this simple hypothesis, we could break away from thinking of ceramide in the singular, which was becoming untenable as ceramide was being implicated in diverse functions through regulation of many of its metabolic enzymes; we simply could not “fit” everything into one simple pathway. Bart-jan Kroesen and Hannie Sietsma, visiting scientists from the Netherlands, provided solid evidence for distinct regulation of long-chain *versus* very long–chain ceramides during apoptosis (69).

At the cell biology level, Besim Ogretmen studied the deacylation/reacylation of ceramide and then implicated ceramide in the regulation of telomerase (70, 71). Charles Chalfant implicated *de novo* synthesized ceramide in the regulation of phosphorylation of the SR proteins (72). He also commenced his work on ceramide kinase and inflammation (with Pettus) (73), which then became the focus of his independent career. Ben Pettus also defined roles for sphingosine kinase in driving production of cytokines and inflammatory mediators (74). Kazuyuki Kitatani developed studies on the salvage/recycling pathway and its regulation, and the involvement of both acid sphingomyelinase and acid glucocerebrosidase (75). The latter was connected to inflammatory responses whose relevance to Gaucher’s disease was investigated in collaboration with the group of Greg Grabowski (76). An offshoot of these studies led Masa Wada to implicate the p38 MAPKδ (77) in breast cancer pathogenesis. Youssef Zeidan connected the formation of PM ceramide (PM-Cer) to the dephosphorylation of ezrin and related proteins (78). In the past few years, this has become a main focus.

We continued our studies on defining targets for ceramide. Katsuya Kishikawa discovered the activation of the phosphatase PP1 by ceramide (79). Kishikawa, Charles Chalfant, Jeff Jones, and Sehamuddin Galadari (a visiting scientist) advanced these studies and also discovered potent inhibitory effects of PA on PP1 (80, 81, 82). David M Perry identified PP2C phosphatase as another target for ceramide *in vitro* (83).

Our chemical biology approaches led by Ala Bielawska and Zdzislaw Szulc resulted in several interesting molecules including lysosomally targeted inhibitors of acid ceramidase and mitochondrially targeted ceramides (84, 85).

### Enzymes and more enzymes

As the biochemistry and cell biology of sphingolipids were rapidly advancing through the work of several labs across the world, I realized we needed to readjust our emphasis, at least temporarily, from the bioactivity of sphingolipids (a lot of cell biology and chemical biology) toward focus on the regulation and function of individual enzymes. I termed the latter the “*enzyme centric approach”* and the former, the “*lipid centric approach”* (67). Focusing on enzymes continues to allow/force us to study one pathway at a time, how it is regulated, and what its functions are. This also brings in the use of advanced cell and molecular biology tools available for genes and proteins.

At this point, we started the very long-term focus on neutral sphingomyelinase 2 (nSMase2). We defined a role for it in cell confluence-induced arrest and roles in regulating adhesion molecules—studies carried out mainly by Chris Clarke and the late Norma Marchesini (86, 87, 88). Bill Wu also defined its regulation by anionic phospholipids (89), Clarke defined its regulation by kinases (which connected it to PKC and TNF) (90), and phosphorylation was shown by the Goldkorn group (91). Motohiro Tani discovered palmitoylation of nSMase2 (92) and determined its localization to the inner leaflet of the plasma membrane and its topology (93). Delphine Milhas studied the regulation of transportation of nSMase2 from the Golgi to the PM (94). Chiara Luberto worked with Glaxo-Wellcome (now GlaxoSmithKline) to develop an inhibitor for nSMase2 (GW4869) (95), which is still widely used in culture and *in vivo*. She also implicated the enzyme in TNF-induced mitochondrial dysfunction.

Samer El-Bawab purified (the old-fashioned way) (96) and cloned (97) neutral ceramidase and defined *in vitro* mechanisms of regulation (98). He and Julnar Usta (visiting scientist from AUB), working with Zdzislaw Szulc (99, 100), conducted structure-activity studies on its substrate preferences and inhibitors that we developed in house. In collaboration with Rick Proia, we generated the KO mouse for this ceramidase (101). Bill Wu identified mitochondrial sphingomyelinase, which showed similar biochemical features to nSMsae2 (102) but appeared to reside primarily in the mitochondria as studied by Vinodh Rajagopalan (103). Hirofumi Sawai worked on another putative sphingomyelinase, nSMase1, and found that physiologically it functioned as a lyso-platelet–activating factor phospholipase C (104). Three MD-PhD students, Youssef Zeidan, Russell Jenkins, and David M Perry, studied acid sphingomyelinase, another key regulator of ceramide formation. Zeidan connected it to regulation by PKC (105). Jenkins studied the regulation of its secretion (106) and maturation (107) and found that the secreted form of the enzyme plays important roles in inducing inflammatory responses (108), work that was advanced by Perry (109). This is of clinical significance as gene therapy to overcome the deficiency of the enzyme in Niemann-Pick disease resulted in significant inflammatory responses (110). Finding a form of the enzyme that would not induce inflammation but correct the metabolic defects of acid sphingomyelinase would thus be of high therapeutic value.

In collaboration with Jackie Kraveka, we worked on dihydroceramide desaturase 1 and defined roles in cell cycle regulation (111). We also discovered that the chemotherapeutic fenretinide (a lipid amide and an analog of retinoic acid), directly inhibited the enzyme *in vitro* and in cells (112). Jola Idkowiak-Baldys and Aintzane Apraize showed regulation of dihydroceramide desaturase 1 by oxidative stress (113).

### Yeast work

We continued our yeast work, and this netted us the identification of Isc1, the homolog of mammalian neutral sphingomyelinases, through the efforts of Hirofumi Sawai (114). Yasuo Okamoto studied the interaction of Isc1 with anionic phospholipids and defined the domain required for this interaction (115). Silvia Vaena-de Avalos studied the regulation of Isc1 by cardiolipin (116) and, in a collaboration with William Dowhan (117), connected activation of Isc1 in the postdiauxic shift to the phosphatidylglycerol/cardiolipin pathway in mitochondria (118). Building on this work, Hiroshi Kitagaki defined the regulation and action of Isc1 in yeast mitochondria and defined a role for the enzyme in connecting mitochondria to the regulation of nuclear genes involved in metabolism of nonfermentable carbon (119, 120). We also discovered several key functions for Isc1 in regulation of the diauxic shift, in morphogenesis, and in response to DNA damage. Nabil Matmati connected Isc1 to hydroxyurea, showing that it is required for survival (121).

Building on his discovery of regulation of the *de novo* pathway in yeast by heat stress, Gary Jenkins and then Ashley Cowart defined a role in the transient cell cycle arrest and in the dephosphorylation of a subset of RNA-binding proteins (122, 123). Cowart also performed microarray analysis of the heat stress response when it was still a novelty, and the results confirmed roles for sphingolipids in stress responses and cell cycle regulation and revealed additional regulation of amino acid metabolism, cell wall, and transport (124). A few years later, David Montefusco implicated *de novo* synthesized sphingoid bases in a feedback process, regulating the induction of the serine deaminase Cha1 (125). Cowart also showed that bioactive sphingolipids are involved in regulation of cell cycle arrest in response to heat stress and in formation of P-bodies (126). Other laboratories extended the functional significance of the *de novo* pathway. A neat new direction occurred as a result of a strong collaboration with Eberhard Voit, a pioneer of modeling of biochemical pathways, in work driven by Fernando Alvarez, a joint postdoc. This resulted in modeling the yeast *de novo* pathway, which was published in Nature (127). In another combined bioinformatic/biochemical approach working in collaboration with Xinghua Lu, Montefusco was able to assign specific correlations and functions to distinct ceramide species (128).

### Not forgetting PKC

We also continued research on PKC. In an elegant set of studies, Kevin Becker identified a novel translocation of some PKCs to a perinuclear region (129) and showed the involvement of phospholipase D in this process (130). Jola Idkowiak-Baldys continued these studies and defined a role for sustained activation and translocation of PKC in sequestering a subset of recycling cargo (131), and, in collaboration with John Raymond, she defined its role in response to sustained G protein receptor stimulation (132). Mohammad El Osta identified substrates for PKC action at the PM *versus* substrates requiring this novel translocation (133).

### The Charleston Ceramide Conference gets started

**Figure 6:**
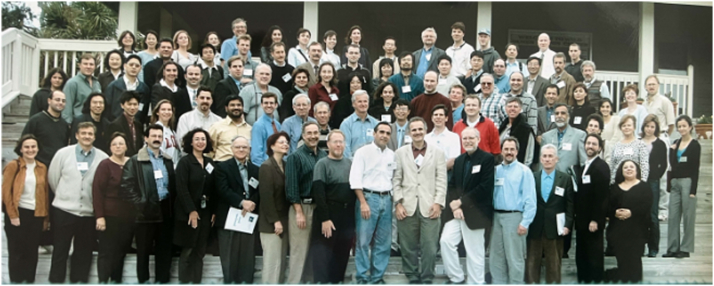
**The Inaugural Charleston Ceramide Conference (later, the international Ceramide Conference or iCC) held in Wild Dunes, Isle of Palms, SC 2001**.

In a desire to bring together the main investigators in the field and their students, we (myself, Lina, Myles Cabot, and a few others) initiated the Charleston Ceramide Conference soon after moving to Charleston in 2001 (Fig. 6). It is still ongoing on a biannual basis as the international Ceramide Conference. While keeping the conference going has required significant effort, it is now run by a team of several investigators. Importantly, it still maintains its original distinctive mission to mostly cater to students and the more junior investigators, and it has become a very effective vehicle for catching up on the rapidly growing field, for elevating the quality of the work, and for fostering camaraderie and collegiality.

## Stony Brook University (2012–present)

After a nearly 15-year stint at MUSC, Lina and I (and most of the lab group) moved to Stony Brook University, where I took on the job of Cancer Center Director, and Lina became the Dean for Research in the School of Medicine. We both had opportunities to build institutional research programs while maintaining our own research activities, which continued to grow and evolve. There is always a thrill and some apprehension in moving to a new environment, and I find it takes me a year or so to get the lay of the land, both at work and at home. I immersed myself in building a cancer center from scratch, with a wide scope encompassing basic, translational, clinical, and epidemiologic research, as well as dealing with the clinical programs, fund raising, and other administrative aspects.

Amid all that, I still carved time for the lab, which by that time had matured with strong momentum and a wonderful small group of collaborators who moved with us, (Chiara Luberto, Cungui Mao, Chris Clarke, Daniel Canals, Nabil Matmati, Michael Airola, Ashley Snider), some for the second time, and who were joined by a new class of graduate and postdoctoral students (Fig. 3, *upper left*). The field of sphingolipid research was blossoming with labs all around the globe driving new insights and tools.

### More focused work on sphingolipids

**Figure 7:**
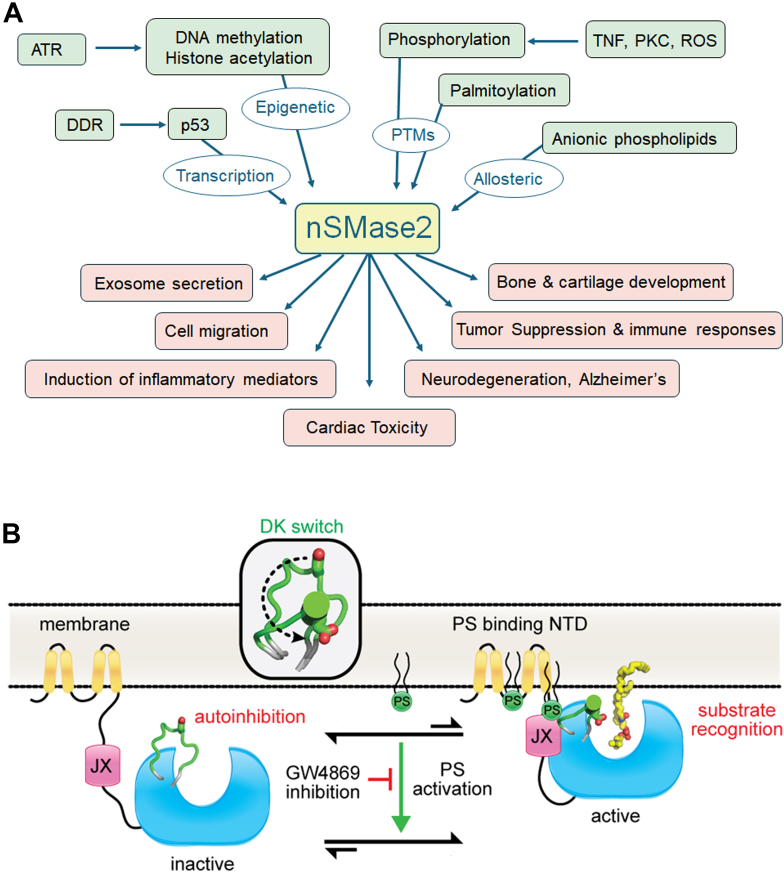
**Regulation, mechanisms, and functions of nSMase2.** Our group has expended significant efforts on studying and understanding nSMase2 at a molecular and cellular level. *A*, summary of what is known about mechanisms regulating nSMase2 (*green boxes*) and participation of nSMase2 in various biologic (cellular and organismal) functions (*pink boxes*). *B*, *cartoon* of biochemical mechanisms of regulation of nSMase2 illustrating allosteric regulation by phosphatidylserine (PS) which binds to two distinct regions in the N-terminal domain (NTD), inducing conformational changes that bring together the Juxta membrane region (JX) to the hydrophobic helices and the catalytic domain. This translates in relieving autoinhibition of the enzyme and position a key aspartate for catalysis. The inhibitor GW4969 reverses the action of PS. *Panel B* is reproduced from (134). nSMase2, neutral sphingomyelinase 2.

My group became more focused on nSMase2 (Fig. 7) and to a lesser degree, neutral ceramidase, with the crystal structures of both determined (before AlphaFold 3 days) by Micheal Airola (134, 135). Prajna Shanbhogue performed some elegant studies to determine intramolecular mechanisms of regulation of nSMase2 (136). Chris Clarke elaborated on the regulation of nSMase2 by retinoic acid (137) and demonstrated that the enzyme is profoundly regulated by epigenetic mechanisms in breast cancer (138), whereas Achraf Shamseddine, an MD-PhD student, defined the regulation of transcription of nSMase2 by doxorubicin and implicated it in the DNA damage response (139). Another graduate student, Samia Mohammed, continued the work and started implicating nSMase2 in the cardiac toxicity induced by Doxorubicin, in a collaboration led by Chris Clarke (140). Dani Canals, then a postdoc who is now junior faculty, has focused on PM-Cer. He consolidated the role of ceramide in the PM to induce ezrin dephosphorylation and discovered a profound effect of S1P on inducing ezrin phosphorylation (141). He developed an assay to quantitate ceramide specifically in the PM-Cer (142), and then studied its metabolism (143) and cell biologies linked specifically to this pool of ceramide. Through this work, he demonstrated that PM-Cer selectively regulates cell migration and adhesion in a process dependent on protein phosphatase 1α (144), and then he showed that the pathway was activated by sublethal concentrations of the chemotherapeutic Doxorubicin to drive migration (145). He also showed that nSMase2 plays a key role in regulating PM-Cer. These ongoing studies now allow us to dissect goals we had set for ourselves back in the early 1990s with the demonstration of very acute activation of SMases and induction of ceramide at the PM with specific functions.

Our studies also involved ceramide kinase as an outgrowth of our previous studies on acid sphingomyelinase. Ben Newcomb connected acid sphingomyelinase to the subsequent action of ceramide kinase and then to the production of chemokines (146). In other studies, Monica Garcia-Barros showed that neutral ceramidase plays an important role in carcinogen-driven colon cancer (147), and Nicolas Coant showed regulation of mTOR by the enzyme (148). He is now engaged in developing inhibitors for it.

Our lab has continued to advance methods and tools to probe sphingolipid metabolism. Over the years, we developed analogs of sphingolipids, targeted sphingolipids, and inhibitors of enzymes. We had developed the initial sphingolipidomic approaches and built on this work (*e.g.* studies by Nadia *Rana*, a post doc). More recently, Justin Snider advanced methods for flux analysis (149), revealing complexity in regulation of sphingolipids by Doxorubicin (150). Wataru Sakamoto elaborated compartmentally-targeted bacterial sphingomyelinases and ceramidases to probe the subcellular specific functions of sphingomyelin, ceramide, and sphingosine (151, 152). Allen Lee recently developed a simple method to probe ceramide metabolism in the Golgi and to evaluate the action of relevant inhibitors (153).

### Continued yeast work

While at Stony Brook, we also continued our work on yeast. Nabil Matmati, who moved with us from Charleston, continued working on HU, showing it selectively employs C18:1 phytoceramide to regulate survival in response to HU (154). Matmati and Bachar Hassan then defined a role for Isc1 and its downstream biochemical target the phosphatase subunit CDC55 in regulating the spindle check point (155). Jihui Rhen, developed advanced LC-MS/MS to study synthesis and metabolism of SPLs in yeast. She found that the very first product of *de novo* synthesis, 3-ketodihydrosphingosine, thought to be fleeting, was measurable at levels comparable to dihydrosphingosine (156). She also implicated Tsc3, a small subunit of SPT, in the selective production of deoxysphingolipids (157). Her latest work has focused on the profound effects of the ORM proteins, regulators of SPT, on sphingolipid synthesis, identifying a novel regulatory module involving SPT, ORMs, sphingoid bases and the kinase Ypk1 (158).

### PKC comes home

We continued our work on PKC, which became more focused on defining the function of PKCα in cancer signaling, especially relating it to the AKT/mammalian target of rapamycin (mTOR) pathway. Mengling Liu linked sustained activation of the classical PKC isoenzymes to activation of phospholipase D and then mTOR (159). Mohamed Salama implicated PKCα in the function of mutant epidermal growth factor receptor (mEGFR), a key oncogenic driver in lung adenocarcinoma, and he also connected it to mTOR (160). Mojtaba Sadeghi, with support from Salama, established that the mEGFR was actually not fully “intrinsically” active, as touted, but was quite defective in ligand-free and ligand-activated signaling. It shows biased signaling as it primarily couples to phospholipase C γ and hence to PKC, and it becomes dependent on both for its oncogenic signaling, with PKCα assuming a critical role in activating the Akt/mTOR pathway and in mediating anchorage-independent growth and survival of mEGFR cells (161). In the most recent set of studies, Sadeghi has found that PKCα plays a key role in the generation of drug-tolerant persisters, which are cells that are reversibly resistant to the action of therapeutics but are thought to serve as a platform for the emergence of irreversible (genetically) resistant cells. To me this is a very gratifying culmination of 4 decades of working on PKC, during which time very little rigorous cell biology has been done to demonstrate important, critical, and specific functions for PKC isoenzymes. This is not intended to discourage investigators, or to imply that we are always slow in moving forward. Sometimes advances do require new insights and new tools to take a field to the next level.

### An interesting diversion into cancer epidemiology

The cancer leadership role also led me down an unexpected path. Examining a published study on cancer risk that claimed most cancer risk is “intrinsic” and not driven by extrinsic factors, I realized that these critical conclusions were not actually supported by the data. This started a collaboration with Song Wu, a very talented mathematician, and we published what I consider a neat paper in Nature (162) and then other papers showing that most cancers arise due to external risk factors. In this work, we used four distinct approaches to demonstrate that at least 70% of cancer risk is driven by extrinsic factors and exposures. These papers garnered a lot of media attention, and I had my 15 min of fame, with which I was extremely uncomfortable. But the experience reinforced the need for scientists to communicate to the public clearly while not succumbing to the pressures to oversimplify and/or exaggerate.

### Sociologic contributions to science

I have always been interested in the sociology of science, and over the years I have contributed a few position papers, reflecting on: 1) the need to apply scientific methods by scientists when they tackle policy issues (163); 2) the rapid expansion of pools of trainees beyond the ability of the “system” to sustain their career development (164); 3) I suggested the development of a registry whereby undergraduate students attempt to replicate key published results in order to establish facts that are reproducible and on which one can build deeper insights (165). This last is quite pertinent in the current era of big data. There is always the danger of “garbage in, garbage out.” I find it troubling that there are so many results in the literature that are not confirmed, and worse, not confirmable.

## Losing Lina (2019)

Lina’s first encounter with cancer occurred in 2005 when she was diagnosed with colon cancer that was successfully resected. She was fortuitously monitored by measuring carcinoembryonic antigen (CEA) levels (a frequent biomarker of colon cancer, which her cancer did not display). Over a few years, her CEA started slowly increasing, and ultimately, it was traced to a new lung cancer that was diagnosed in 2010 (CEA is an unusual biomarker for lung cancer). This was lung adenocarcinoma with mEGFR, and it was successfully resected with no evidence of recurrence. She passed the 5-year mark in 2015, which is often considered “cure.” But that was not to be. Her cancer recurred in 2017 in the form of a small mass in the dura. This was resected, and she was started on targeted inhibitors for mEGFR to which she initially responded, followed by small recurrences that were successfully irradiated. In August of 2019, however, her disease rapidly progressed. She passed on November 29, 2019.

Everyone who knew Lina appreciated how she would light up the room as she walked in. Lina and I were partners at home as well as in research, and we complemented each other on multiple levels. Lina brought molecular biology expertise and her own intuition as well as a more focused translational point of view. She adopted her students and helped them navigate their scientific growth; many of them (and several of mine) would also turn to Lina for personal advice (Fig. 3, *lower panels*).

Lina was a role model for female scientists, helping them develop and juggle work, family, and life responsibilities. At MUSC, she led the COBRE program in sphingolipids for 12 years: an NIH-funded competitive program aimed at advancing the careers of junior faculty, many of whom were female. A number of her trainees are now leaders in biomedical research.

Lina left an enormous legacy in the study of sphingolipids. We collaborated on several aspects, including the study of apoptosis and its regulation, studies on PKC, and cloning of yeast genes (between our groups, we cloned six out of the 13 yeast genes involved in metabolism of bioactive sphingolipids in yeast). Mark venable, working with Lina at Duke, identified, for the first time, a role for ceramide in the induction of senescence (166), a role buttressed by the subsequent realization that the first eukaryotic gene ever identified to regulate longevity, Lag1, was indeed the main yeast ceramide synthase (167). Stefka Spassieva studied regulation of ceramide synthase (168) and Leah Siskind connected that to the action of the proapoptotic Bak (169). Tom Mullen studied the substrates specificities of the CerSs (170) and their roles in apoptosis (171). In a very elegant study, Can Senkal, working with Lina, discovered the formation of acylceramide as a mechanism to “detoxify” ceramide. They showed that the enzyme DGAT2 is key to formation of acylceramide and that it interacts with CerS and ACSL5 (172).

Lina’s group was mostly focused on studies of sphingosine kinase 1 (SK1). They studied it in migration, inflammation, arthritis, vasculogenesis, and especially cancer and the DNA damage response. They defined mechanisms of regulation in cells and biochemically. We also collaborated on several studies on sphingolipids. Toshi Kawamori showed that the SK1 KO mouse was protected from experimental colon cancer (173), and Ashley Snider showed that the KO was protected from experimental colitis (174). DeAnna Baker, in collaboration with Gary Gilkerson, showed that the KO was also protected in a model of inflammatory arthritis (175). Korey Johson found that SK1 is highly expressed in many cancers (176), and he defined a role for PKC in the translocation of SK1 to the PM (177). Viviana Anelli defined a role for SK1 in lymphangiogenesis (178) and defined regulation of SK1 by HIF1a (179). Tarek Taha discovered that genotoxic stress induces a p53-dependent loss of SK1 (180), and Linda Heffernan showed that SK1 is required for the generation of thymic lymphoma in the p53 mutant mouse (181). Chris Gault implicated SK1 in the action of the K-Ras on sphingolipids (182), whereas Alexa Orr demonstrated a role for S1P receptor 2 in mediating the effects of epidermal growth factor receptor on ezrin phosphorylation (183). Ashley Snider dissected the roles of SK1 in colitis (184), and Mohamed Salama defined its role in the invasiveness of kidney cancers with VHL mutations (160). Brittany Carroll continued the work on connections between p53 and SK1, implicating caspase 2 in SK1 proteolysis (185). Mohamad Adada implicated SK2 in mediating the effects of epidermal growth factor receptor on ezrin (186) while further connecting SK1 to cellular inflammatory responses (187). Mike Pulkoski-Gross, a graduate student, defined regulation of SK1 by anionic phospholipids and mechanisms involved in translocation (188). Mel-Pilar Espaillat showed that loss of acid ceramidase, specifically in myeloid cells, suppressed neutrophilic infiltration in colitis (189).

Our groups also collaborated on apoptosis studies. Joanna Lea worked on ceramide, cell cycle, and PKC (190, 191), while Helen Birbes, building on work by Peng Zhang (192), showed that mitochondrial ceramide was specifically associated with apoptosis (193).

Sergei Novgorodov defined the effects of long-chain ceramides on mitochondria (194). He also defined a role for the reverse activity of neutral ceramidase in generation of mitochondrial ceramide (195). Nick Schwartz showed that decreased ceramide resulted in mitochondrial dysfunction in Charcot-Marie-Tooth disease type 2F (196).

With Cungui Mao, Lina studied the family of the alkaline ceramidases that they discovered first in yeast and then in human, and they undertook biochemical and cell biologic studies. Mutations in these genes cause hereditary neurologic disorders. The many contributions of Lina’s lab are presented in this review (197).

Lina received many honors and recognitions during her lifetime, including election to American Association of Physicians, American Association for Advancement of Science, and Alpha Omega Alpha Medical Honor Society. She was the Boyle professor at MUSC and a SUNY distinguished professor at Stony Brook. She was on the editorial board of JBC and other journals. An endowed chair is named after her at Stony Brook, for use by the person occupying the position of Dean for Research in the School of Medicine.

Her legacy includes our three children, six grandchildren, and the myriad students, postdocs, junior faculty, and senior faculty that she helped in various capacities.

After losing her, and with the help of Drs. Luberto, Clarke, and Mao, we took on the completion of training of her students and postdocs. They all finished successfully, and they made important contributions. JP Truman published important results on the role of SK1 in the response of cells to serine deprivation (198), leading to the formation of deoxysphinganine, which in turn, he showed, causes loss of SK1, buildup of sphingosine, and activation of *de novo* synthesis of serine (199). Magali Trayssac showed that oncogene (KRas)-induced senescence resulted in inhibition of SK1 and accumulation of dihydroceramides through CerS2, leading to senescence (200). Joe Bonica demonstrated a key role for SK1 in mediating effects of doxorubicin on angiogenesis (201). Fabiola Velazquez, building on Lina’s discovery that loss of p53 leads to activation of SK1, showed that loss or inhibition of SK1 prevented emergence of thymic lymphoma in the p53 KO mouse (202).

## Final thoughts

Four decades of work on these goals illustrates how slowly science can move, especially for a field that started from a very small foundation. When we commenced our studies, our thinking was these goals would be relatively easy to accomplish, perhaps requiring a few years of work on defining the “SM cycle.” However, along the way we began to appreciate how vast the major gaps in knowledge were, and we also encountered increasing complexities. The latter have been discussed more extensively elsewhere (203), but briefly, we found that it was not one ceramide we had to tackle but many ceramides as amply revealed by LC-MS/MS. The different ceramides are increasingly associated with distinct functions and perhaps targets. We (the field) found out there are dozens of mammalian enzymes that work directly on ceramide as either substrate or product, each of which has been shown to potentially regulate ceramide levels. We (again the field) had to identify, clone (and sometimes purify), and characterize the enzymes. These enzymes and the associated lipid substrates and products demonstrate significant subcellular compartmentalization.

My analogy for this complexity is that we started inside an onion trying to peak at the world. Every time we peeled off one layer, there was another one to tackle. Lots of crying along the way!

The good news is that it looks to me that we are dealing with the last layer. We know we have to focus on specific enzymes, lipids, compartments, biologies, targets, and mechanisms. The field is moving much faster now with much clearer directions and goals. We are in a phase of resolution of complexity. Paradoxically, it is easier to understand and even embrace a field when it enters this stage of resolution despite its complexity. For example, investigators who do not work on eicosanoids are comfortable knowing and appreciating that there are many enzymes and bioactive eicosanoids with distinct targets and cellular functions.

**Figure 8:**
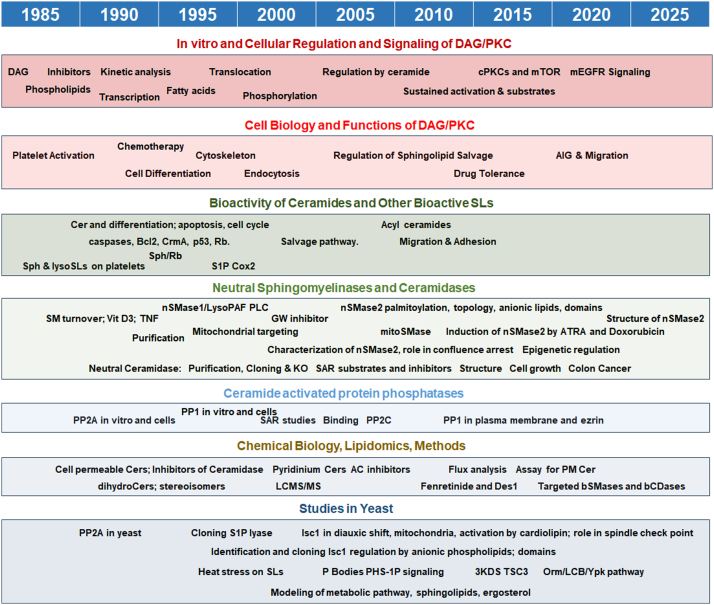
**Timeline of evolution of projects.** This diagram shows progress over the years on DAG/PKC and sphingolipids. DAG, diacylglycerol.

In our case, the evolution of projects (Fig. 8) followed a trajectory whereby we started with finding what we thought was a basic module of sphingolipid signaling at the PM. But we were stumped by the lack of biochemical, chemical, and molecular tools to probe the pathway. We then embarked on a long journey to develop such tools and insights, and on the way, we explored and surveyed the biologic domains of sphingolipids and their regulation—advances that seemed to relentlessly lead us to more complexity. But as of the last few years, we have started making significant advances on the original paradigm of PM signaling, enabled by developing a very powerful assay to measure PM-Cer followed by studying metabolism of ceramide at the PM. These developments are rapidly advancing our understanding of the functions, signaling, and mechanisms of ceramide and nSMase2 at the PM, which has become much more tractable and rewarding.

### Biology, biology everywhere but few mechanisms to hang on

With due apology to Coleridge, this is obviously a great exaggeration, but in my estimation, the field now enjoys an abundance of biologic insights into the various roles of bioactive sphingolipids; it is difficult to fully study a specific cell biology without stumbling into lipids. Therefore, the most pressing challenges for us are in identifying specific mechanisms that regulate specific enzymes and even more so identifying direct targets (mostly proteins) for specific bioactive sphingolipids, focusing on different ceramides (or at least subclasses of ceramide). More sophisticated tools are required to define these protein targets, study their interactions, and, critically, demonstrate their specificities.

Luckily, more precise and enabling tools and approaches are now becoming available along with more insights that are driving faster progress. LC-MS/MS has been quite enabling. More recently MALDI imaging is allowing for detailed study of sphingolipids at the tissue level and even at the single cell level. Newer and more precise chemical tools to probe the pathways and lipid protein interactions are also being developed at an increasing pace. Finally, one should not underestimate the impact of the accumulating insights accruing to the field. Imagine having a new jigsaw puzzle and only a few pieces already inserted on a mostly blank board. It would be very hard to place a new piece. On the other hand, when the puzzle starts to show domains and structures, it becomes much easier to insert new pieces and the whole/overall picture begins to reveal itself. I believe we are at this stage.

## Conflict of interest

The authors declare that they have no conflicts of interest with the contents of this article.
